# Biofilm formation and antimicrobial resistance pattern of uropathogenic *E. coli* ST131 isolated from children with malignant tumors

**DOI:** 10.1038/s41429-024-00704-8

**Published:** 2024-03-04

**Authors:** Noha Anwar Hassuna, Eman M. Rabea, W. K. M. Mahdi, Wedad M. Abdelraheem

**Affiliations:** Medical Microbiology and Immunology Department, Faculty of Medicine, Minia University, Minia, Egypt

**Keywords:** Antimicrobial resistance, Biofilms

## Abstract

The multidrug-resistant clone identified as *Escherichia coli* sequence type 131 (*E. coli* ST131) has spread world-wide. This study sought to ascertain the frequency and biofilm formation of *E. coli* ST131 isolated from children with various malignancies. A total of 60 uropathogenic *E. coli* (UPEC) isolates from children without cancer and 30 UPEC isolates from children with cancer were assessed in this study. The microdilution method was used to investigate the sensitivity of bacteria to antibiotics. The microtiter plate (MTP) approach was used to phenotypically assess biofilm formation. The *las*R, *pel*A, and *lec*A biofilm-encoding genes were detected by PCR in biofilm-producing isolates of *E. coli*. Thirty-seven out of 90 *E. coli* isolates were found to be ST131 (41.1%), with 17 (56.7%) from cancer-affected children and 20 (33.3%) from children without cancer, respectively (*P*-value = 0.036). The frequency of antimicrobial resistance was higher in ST131 strains were compared to non-ST131 strains and when they were isolated from healthy children vs. those who had cancer. In contrast to non-ST131 isolates, ST131 isolates were more biofilm-producers. There was a significant difference between the percentage of biofilm producers between the 22 (100%) ST131-O16 isolates and the 13 (86.7%) ST131-O25b isolates (*P*-value = 0.04). Children with cancer are more likely than children without cancer to develop biofilm forming *E. coli* ST131, the latter having a higher profile of antibiotic resistance. Interestingly, *E. coli* ST131 isolates from non-cancer patients had higher levels of overall antibiotic resistance and while more *E. coli* ST131isolates from cancer patients formed biofilms.

## Introduction

Multidrug resistant *E. coli* ST131 clone globally and is involved with infections that are antibiotic resistant [1]. Multi-locus sequence typing (MLST) allowed for the 2008 identification of the ST131 *E. coli* clonal group, which had previously been unknown [2]. ST131 strains primarily belong to phylogenetic group B2 and, to a lesser extent, group. *E. coli*-ST131 primarily belongs to the serotype O25:H4, which has the O25 type O25b, as determined by O and H antigens. Moreover, ST131 isolates of the serotype O16:H5 have been found many countries [3]. In addition to typically having extended-spectrum -lactamase (ESBL) genes such as *bla*_CTX-M-15_, almost all *E. coli* ST131 strains are resistant to fluoroquinolones and antibiotic resistant *E. coli* is increasing globally [3]. Pathogenic strains of *E. coli* aggregate and become challenging to eliminate because of biofilm development. Several studies have examined the capacity of the ST131 strain to develop biofilm [4, 5]. Patients with cancer are more disposed to bacterial infections in general, and uropathogenic *E. coli* urinary tract infections in particular (UPEC). Damage to the normal anatomical barriers, surgical operations, chemotherapy, radiation, nutritional variables, and the growing use of medical devices are among the factors that raise the risk of infection in these patients [6]. Therefore, the purpose of this study was to assess the frequency and capacity of uropathogenic *E. coli* ST131 to form biofilms when isolated from both cancer-affected and cancer-unaffected children.

## Methods

The present study included 245 urine samples. 90 samples were collected from children with cancer attending the Pediatric Department of Minia National Oncology Institute (case group) and 155 samples were collected from children without cancer attending the Pediatric outpatient clinics of Minia University hospital (control group) during the period from October 2020 till October 2021. Children aged 2–18 years with clinical symptoms and signs of urinary tract infection (fever, dysuria, frequency, neutropenia or with bacteriuria >10^5^ organisms/ml) were included. Phenotypic and genotypic characterization was carried out in the Microbiology and Immunology Department, Faculty of Medicine, Minia University.

### Bacterial isolation

*E. coli* isolates were identified and isolated according to the standard methods using Gram staining, colony morphology (on MacConkey and EMB agar) and standard biochemical tests (IMVC) [4]. Confirmed *E. coli* isolates were stored in trypticase soy broth with sterilized 15% glycerol at −20 °C.

### Antibiotic susceptibility testing

All *E. coli* isolates were examined for antibiotic susceptibility by the micro-dilution method following the Clinical and Laboratory Standards Institute (CLSI) recommendations [5].The panel of antibiotics was as follows: cefazolin, ampicillin-sulbactam, cefoxitin, levofloxacin, meropenem, tetracycline, ceftazidime and nitrofurantoin.

#### Detection of biofilm formation

The *E. coli* isolates were tested phenotypically for their ability to form biofilm by the Microtiter Plate Method (MTP), and genotypically by detection of biofilm encoding genes by PCR.

#### Microtiter Plate method (MTP)

This quantitative test is considered the gold-standard method for biofilm detection. Briefly, 3–5 of isolated pure colonies were suspended in Muller Hinton broth (MHP) with 2% sucrose and then incubated for 18 h at 37 °C in a stationary condition. After adjusting the turbidity of bacterial broth to 0.5 McFarland standard, 200 μl of bacterial broth was inoculated into a sterile MTP wells except the wells of the last column that was used as a negative control containing sterile MHP. The inoculated plates were incubated for 48 h. The contents of wells were decanted and the remaining bacteria in the wells were washed with saline. The bacteria in the wells were stained using 150 μl of crystal violet (0.1%) for 15 min at room temperature. The stain was aspirated by pipette and washed with water. One hundred fifty μl of 95% ethanol was pipetted into each well gently and the wells were covered to minimize evaporation covered to minimize evaporation. The results were interpreted by an ELISA reader at 620 nm. The interpretation and grading (weak- moderate and strong) of biofilm production was done according to the criteria of Stepanovic et al. [6].

### DNA extraction

DNA extraction used overnight broth culture of all *E.coli* isolates. 1.5 ml of bacterial broth was pipetted into sterile microtubes. The microtubes were centrifuged at 5000 × *g* for 3 min. The supernatant was discarded, and the pellet was suspended in 200 μl molecular biology-grade water and mixed by pipetting. The tubes were boiled at 100 °C in a water bath for 14 min; cooled quickly on ice for 20 min, followed by centrifugation for 3 min at 5000 × *g*. Two hundred μl of the supernatant containing DNA was put into a newly labeled tube for PCR analysis.

#### Detection of ST131 *E. coli* isolates

ST131 *E. coli* isolates were identified by the presence of *pab*B or *trp*A genes using multiplex PCR. ST131-O16 was identified by being positive for the *trp*A gene and ST131-O25b were identified by those positive for the *pab*B gene.

#### Detection of carbapenem resistance genes

All phenotypic carbapenem-resistant *E. coli* (CRE) isolates were molecularly tested for the presence of the following carbapenem -resistant genes: *bla*_VIM_, *bla*_NDM_, *bla*_KPC_, and *bla*_IMP_ genes using multiplex PCR.

Multiplex PCR was carried out in 50 µl reactions containing 25 µl hot start 2x Taq DNA Polymerase Master Mix, 6 µl of the extracted DNA and 10 pmol of each of the forward and reverse primers. The reaction was completed by nuclease-free water. The PCR amplification was performed with an initial denaturation at 94 °C for 10 min, followed by 30 cycles of denaturation for 5 s at 94 °C and annealing for 20 s followed by a final extension for 5 min at 72 °C.

#### Detection of biofilm encoding genes

All *E. coli* were molecularly tested for the presence of *las*R*, pel*A*, and lec*A biofilm genes by conventional PCR. PCR reaction was performed in 25 μl of reaction mixture containing 3 μl of extracted DNA, 12.5 μl Taq PCR Master Mix, and 100 pmol (1 μl) of each primer [7–9]. PCR conditions for the amplification step were: denaturation at 94 °C for 1 min, annealing was different according to the tested gene, and extension at 72 °C for 1 min. Cycling was followed by a final extension at 72 °C for 10 min. Primer sequences, annealing temperatures and amplicon sizes of all study genes were presented in Supplementary Table S1.

#### Statistical analysis

SPSS Version 20.0 statistic software was utilized for carrying out the statistical analysis (SPSS Inc., Chicago, IL, USA). Chi-squared tests were performed for categorical data. A two-tailed *P*-value of <0.05 was considered statistically significant.

## Results

Out of 245 urine samples, 166 (67.7%) demonstrated positive bacterial growth, while 79 (32.3%) did not. *E. coli* was found in 90 out of 166 isolates (54.2%), and other bacteria were found in 76 (45.8%) isolates. Female children were more likely to have *E. coli* isolates than male children, with 65 (72.2%) *E. coli* strains being found in female samples and 25 (27.8%) in male samples (*P*-value 0.01).

### Distribution of *E. coli* ST131 among children with and without cancer

Molecular identification of *E. coli* ST131 isolates was done by multiplex PCR for *pab*B and *trp*A genes (Supplementary Fig. S1). Out of 90 *E. coli*, 37 (41.1%) isolates were identified as ST131.The distribution of ST131 isolates was as follow: 17 strains were isolated from children with cancer (56.7%), and 20 strains were isolated from children without cancer (33.3%) with significant difference (*P-*value = 0.036).

**Fig. 1:**
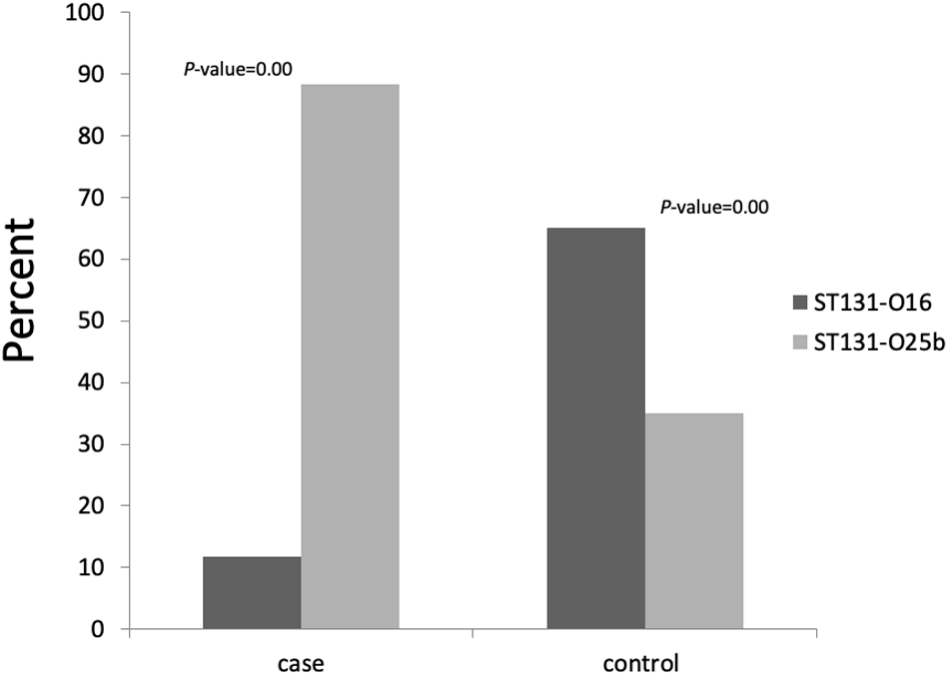
The distribution of ST131clades among case and control isolates. Total number of case and control ST131 isolates was 17 and 20 respectively

ST131 variants were distributed as follow: 15 (40.5%) isolates were ST131-O25b clade *pab*B (ST131-O25b) and 22 (59.5%) isolates were ST131-O16 clade *trp*A (ST131-O16). ST131-O16 was the predominant type among children without cancer (13/20, 65%), while ST131-O25b was the predominant type among children with cancer (15/17, 88.2%) with significant difference (*P*-value = 0.00) (Fig. 1).

### Antimicrobial susceptibility testing

Children without cancer (control isolates) exhibited higher antimicrobial resistance than isolates from children with cancer, with a significant difference in sensitivity to ampicillin-sulbactam, cefoxitin, ceftazidime, and meropenem (*P*-value 0.05) (Table 1). With a significant difference in ampicillin-sulbactam, cefoxitin, levofloxacin, and meropenem (*P*-value 0.05), ST131 isolates had higher antimicrobial resistance than non-ST131 isolates (Table 1). The ST131-O25b and ST131-O16 clades’ antimicrobial resistance patterns to all drugs did not differ significantly (*P*-value 0.05).All phenotypic carbapenem resistant *E. coli* (CRE) isolates were molecularly tested for the presence of the following carbapenem-resistant genes: *bla*_VIM_, *bla*_NDM_*, bla*_KPC_, and *bla*_IMP_ genes by multiplex PCR (Supplementary Fig. S2)*.*

**Table 1:** Antimicrobial resistance among *E. coli* isolates

Antibiotic	Case isolates (%) *N* = 30	Control isolates (%) *N* = 60	*P-*value	ST131 (%) *N* = 37	Non-ST131 (%) *N* = 53	*P-*value
Cefazolin	29 (96.7%)	59 (98.3%)	0.912	37 (100%)	51 (96.2%)	0.522
Ampicillin-sulbactam	12 (40%)	40(66.7%)	0.038	32 (86.5%)	20 (37.3%)	0.042
Cefoxitin	2 (6.7%)	56 (93.3%)	0.000	28 (75.8%)	30 (56.6%)	0.04
Ceftazidime	18 (60%)	57 (95%)	0.000	32 (86.5%)	43 (81.1%)	0.62
Levofloxacin	26 (86.7%)	50 (83.3%)	0.681	35 (94.6%)	41 (77.4%)	0.02
Nitrofurantoin	21 (70%)	52 (86.7%)	0.06	32 (86.5%)	41 (77.4%)	0.23
Tetracycline	25 (83.3%)	51 (85%)	0.837	33 (89.2%)	43 (81.1%)	0.29
Meropenem	8 (26.7%)	27 (45%)	0.043	19 (51.4%)	16 (30.1%)	0.034

Thirty-two (91.4%) CRE isolates harbored *bla*_VIM_ gene, while *bla*_IMP_ gene was detected in 4 (11.4%) CRE isolates*. bla*_NDM_ and *bla*_KPC_ genes were identified in 5 (14.5%) and 2 (5.7%) isolates respectively. More than one gene was detected in 6 (17.1%) CRE isolates. The distribution of carbapenem-resistant genes was higher in ST131 than non-ST131 with a significant difference in *bla*_IMP_ (*P*-value = 0.041) (Table 2). The distribution of carbapenem-resistant genes did not significantly correlate with the origin of the isolates.

**Table 2:** Distribution of carbapenem resistance genes among carbapenem resistant *E. coli* isolates

Strain ID	Type of isolate	Meropenem MIC	Genetic profile			
			_VIM_	_IMP_	_KPC_	_NDM_
C1	Non-ST131	128	−	−	−	+
C12	ST131-O16	128	+	−	−	+
C16	ST131-O16	32	+	−	−	−
C18	ST131-O16	32	+	−	−	−
C68	ST131-O25b	16	+	−	+	+
C67	ST131-O16	256	+	−	−	−
C71	Non-ST131	128	+	−	−	−
C66	ST131-O16	8	+	+	−	+
K2	ST131-O16	512	+	−	−	−
K11	Non-ST131	16	+	−	−	−
K16	ST131-O16	256	+	−	−	−
K17	ST131-O16	256	+	−	−	−
K22	ST131-O16	16	+	−	+	+
K23	Non-ST131	512	+	−	−	−
K26	Non-ST131	16	−	−	−	−
K29	Non-ST131	128	+	+	−	−
K32	ST131-O16	128	+	−	−	−
K43	Non-ST131	16	+	−	−	−
K52	ST131-O16	256	+	−	−	−
K55	ST131-O25b	64	+	−	−	−
K60	ST131-O25b	16	+	−	−	−
K73	ST131-O25b	256	+	−	−	−
K74	ST131-O16	16	+	−	−	−
K90	Non-ST131	128	+	−	−	−
K115	ST131-O16	128	+	−	−	−
K117	Non-ST131	128	+	−	−	−
K130	Non-ST131	64	+	−	−	−
K131	Non-ST131	256	+	−	−	−
K116	Non-ST131	16	+	−	−	−
K28	Non-ST131	8	+	+		
K150	Non-ST131	64	+	−	−	−
K153	Non-ST131	256	+	−	−	−
K147	Non-ST131	16	+	−	−	−
K152	ST131-O25b	16	−	+	−	−
K155	ST131-O25b	32	+	−	−	−

C: case isolate from children with cancer. K: control isolate from children without cancer

### Biofilm formation

**Fig. 2:**
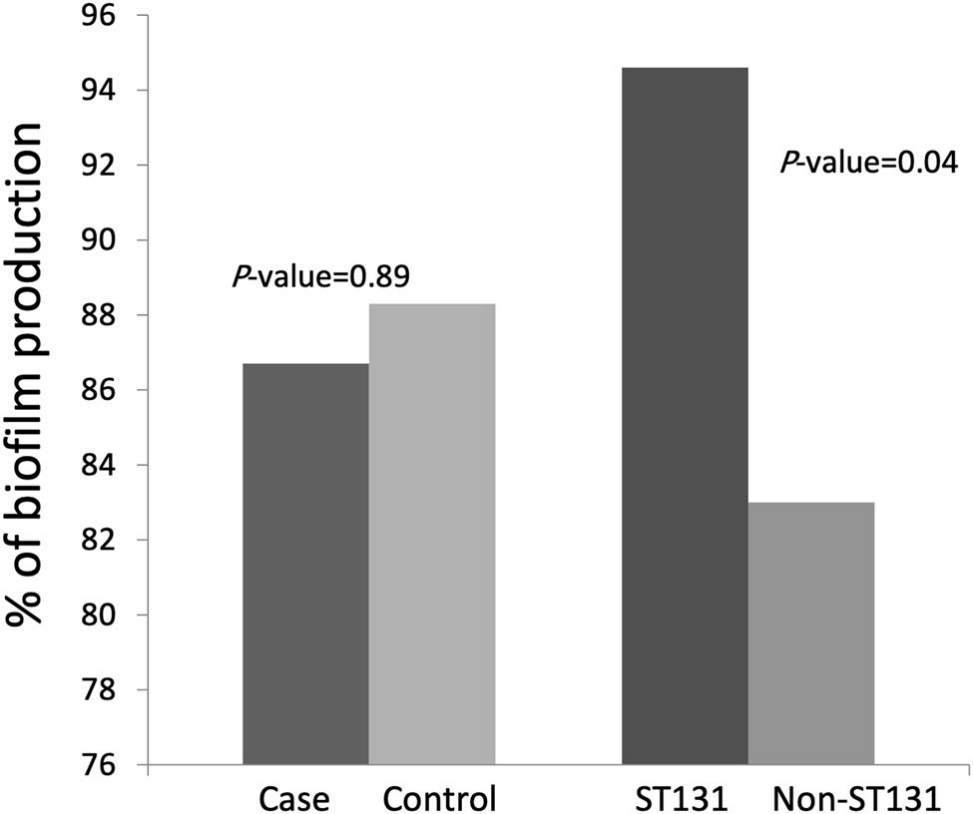
Percent of biofilm production among case and control isolates; ST131 and non-ST131 by MTP method, *P*-value < 0.05 was considered statistically significant

Biofilm was detected in *E. coli* isolates using MTP (Supplementary Fig. S3). Out of 90 *E. coli* isolates; 79 isolates were identified as biofilm-producers by MTP method. *E. coli* biofilm producers were categorized into 3 categories; 21 (23.3%) weak, 39 (43.3%) moderate and 19 (21.1%) strong biofilm producers, according to the amount of biofilm formed. Though not statistically significant (*P-*value > 0.05) the frequency of biofilm producers was higher in the control *E. coli* isolates than in the case isolates (Fig. 2). With a significant difference (*P*-value = 0.04), ST131 strains produced more biofilm than non-ST131 bacteria did. A significant difference between the biofilm-producing abilities of all ST131-O16 isolates (100%) and 13 ST131-O25b isolates (86.7%; *P*-value = 0.04) was found (Fig. 2).

Biofilm-producing *E. coli* isolates phenotypically detected by MTP were tested for the presence of *las*R*, pel*A*, and lec*A (Supplementary Fig. S4). The *las*R*, lec*A *and pel*A genes were detected in all biofilm-producing *E. coli* isolates (100%). On the other hand, *las*R *and pel*A genes were not detected in the non-biofilm producing isolates while *lec*A gene was detected in 41% of the non-biofilm producing isolates.

### Correlation between biofilm production and antibiotic resistance

Regarding levofloxacin, nitrofurantoin, and meropenem, biofilm producers were found to have significantly higher levels of antibiotic resistance than non-producers (*P*-value: 0.01, 0.00 and 0.04 respectively) (Table 3).

**Table 3:** Correlation between antibiotic resistance and biofilm formation

Antibiotic	Biofilm producer 79(%resistance)	Non-biofilm producer 11(%resistance)	*P-*value
Levofloxacin	69 (87.3%)	7(63.6%)	0.012
Tetracycline	67 (84.2%)	9 (81.8%)	0.798
Nitrofurantoin	66 (83%)	7 (63%)	0.00
Meropenem	32 (40%)	3 (27%)	0.04
Cefazolin	79 (100%)	9 (81.8%)	0.59
Ampicillin/Sulbactam	46 (58.2%)	6 (54%)	0.39
Cefoxitin	51 (64%)	7 (63.6%)	0.88
Ceftazidime	67 (84.2%)	8 (72.7%)	0.68

## Discussion

Children with malignancies who undergo cancer therapy have a greater risk of infection due to neutropenia and immunosuppression. Urinary tract infection is one of the most common bacterial infections in children [10]. More than 90% of community-acquired UTI cases, which have high rates of morbidity and mortality and higher healthcare expenditures, are caused by pathogenic *E. coli* [11]. In this study, *E. coli* was isolated from 33.3% of the urine samples taken from children with cancer included. El-Mahallawy et al. identified a similar frequency of UPEC among cancer patients in Egypt [12]. Very few studies have been conducted on the frequency of ST131 in uropathogenic *E. coli* isolates [13]. In one study, Bulut et al., reported similar frequency of ST131 *E. coli* among children [14]. There was no discernible difference between the ST131 isolates and the 13 non-ST131 isolates that were recovered from children being treated for cancer. These results are similar to those reported by Muller et al., who found no obvious variation in the distribution of ST131 and non-ST131 among cancer patients [7]. The frequencies of isolates from the ST131-O16 and ST131-O25b clades were, respectively, 59.5 and 40.5%. Different frequencies of ST131-O25b (77% and 78.6%, respectively) and ST131-O16 (20% and 21.4%, respectively) were reported by Alghoribi et al. in Saudi Arabia, and Hefzy and Hassuna in Egypt [8, 9]. This discrepancy can result from the different age groups and sample size as well as the differences seen in different medical centers. ST131-O16 was the most common type in the current study’s control isolates (65%), while ST131-O25b predominated among isolates from cancer cases (88.2%). Our findings imply that the *E. coli* ST131-O25b lineage may be the predominant *E. coli* ST131 type in cancer-affected children. A substantial difference in ampicillin-sulbactam, cefoxitin, levofloxacin, and meropenem resistance was found between ST131 isolates and non-ST131 isolates (*P*-value < 0.05). This rate was comparable to that reported by Morales-Barroso et al. and Banerjee et al. [15, 16]. In our study, ST131 isolates were 100% resistant to cefazolin, 86.5% resistant to ampicillin-sulbactam, 86.5% resistant to cefoxitin, ceftazidime, nitrofurantoin, and levofloxacin, 89% resistant to tetracycline, and 51% resistant to meropenem. These results were higher than those reported in Egypt by Hefzy and Hassuna [9]. This suggests that ST131 *E. coli* isolates are becoming increasingly resistant to the commonly used antibiotics. Intriguingly, isolates from the control group exhibited higher levels of antibiotic resistance than isolates from the case group. This might be because cancer patients are subjected to stringent rules for antibiotic prescription, whereas control group patients may use antibiotics empirically and needlessly. These findings demand that the misuse of antibiotics among children in the community be addressed properly. The existence of established genetic factors accounted for a considerable portion of in vitro resistance. When carbapenem resistance genes were tested, the majority of CRE isolates (91.4%) had the *bla*_VIM_ gene. Khalifa et al. showed a lower prevalence of the *bla*_VIM_ gene among Enterobacterales in Egypt [17]. Even though *bla*_KPC_ was found to be the primary carbapenem resistance gene in several areas, our study found it in only 5.7% of the isolates, which is lower than other studies conducted in Egypt [18, 19]. Seventy-nine isolates (87.8%) were found to produce in-vitro biofilms using MTP, which was comparable to a previous report in Egypt by Elsayed Gawad et al. [20]. Even though ST131’s epidemiology has been the subject of numerous reports, only a small number of studies have focused on the strain’s capacity to form biofilms. In the current study, ST131 isolates produced biofilms at a rate of 94.6%, which was substantially higher (*P*-value = 0.04) than the biofilm production rate of non-ST13 isolates (83%). This was in agreement to Sarkar et al., who found that ST131 isolates can show considerable changes in biofilm development [21]. Among the ST131 isolates, 100% of O16-ST131 and 86.7% of O25b demonstrated the capacity to produce biofilms. In our study, *las*R*, lec*A, and *pel*A genes were detected in all phenotypically biofilm-producing *E. coli* strains (100%). Several studies indicated that *las*R*, lec*A, and *pel*A genes play an important role in *P*. aeruginosa biofilm formation [22–26], but no previous research has studied the role of such genes in *E. coli* biofilm formation.

Levofloxacin, nitrofurantoin, and meropenem resistance was substantially higher in biofilm isolates compared to non-biofilm isolates (*P*-value <0.05). Similar findings were reported by Cepas et al. and Pompilio et al., who found that *E. coli* biofilm-producers were more resistant to ceftazidime and ampicillin than isolates that did not develop biofilms [27, 28].

There are some limitations encountered with this research and could be addressed in future work such as that the study only comprises isolates collected over one year and at a single medical center.

## Conclusion

The ST131 clone of *E. coli*, which has a high propensity for biofilm formation and antibiotic resistance, has been increasing among children generally as well as among children who have cancer specifically. All ST131 *E. coli* isolates showed an increase in resistance to routinely used antibiotics. When planning molecular epidemiological surveys to ascertain the processes behind the observed resistant phenotypes of the ST131 clone of *E. coli*, it is important to consider the range of distinct resistance genes discovered in this study.

### Supplementary information

Figures

Supplementary tables

## Data Availability

All data generated or analyzed during this study are included in this article and Supplementary files.
