# Supplementary material for: Biofilm formation and antimicrobial resistance pattern of uropathogenic E. coli ST131 isolated from children with malignant tumors
Source: J Antibiot (Tokyo). 2024 Mar 4;77(5):324–30. doi: 10.1038/s41429-024-00704-8 (PMC11058308; doi:10.1038/s41429-024-00704-8)

Supplementary figure S1

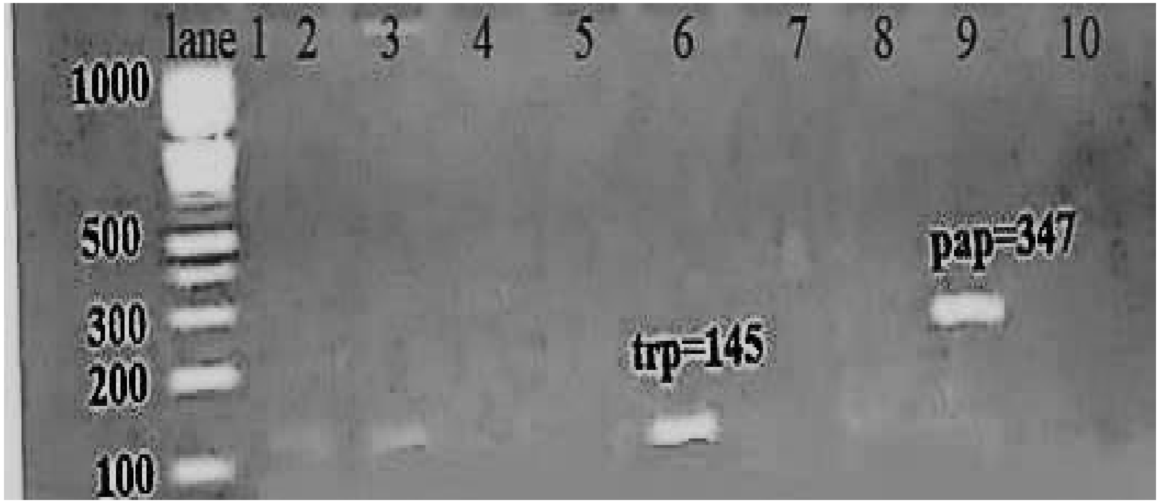

Supplementary figure S2

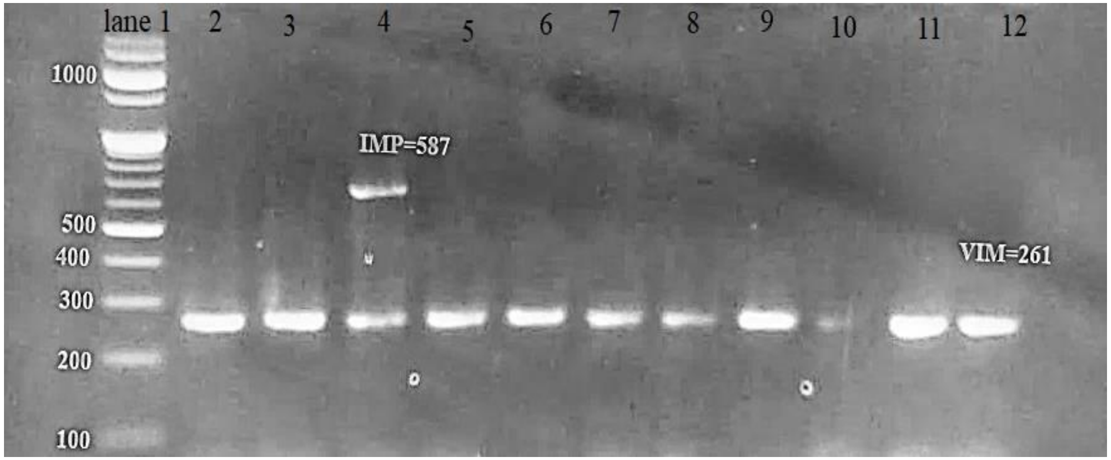

a

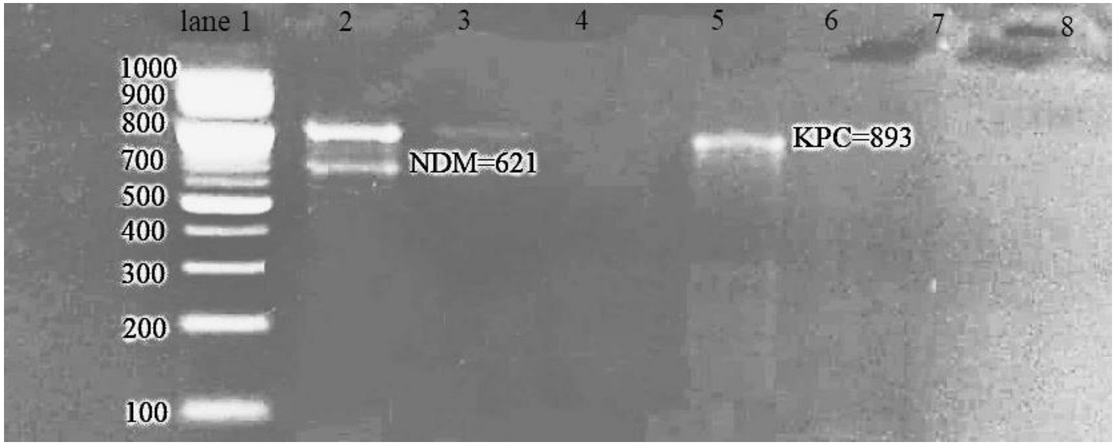

b

Supplementary figure S3

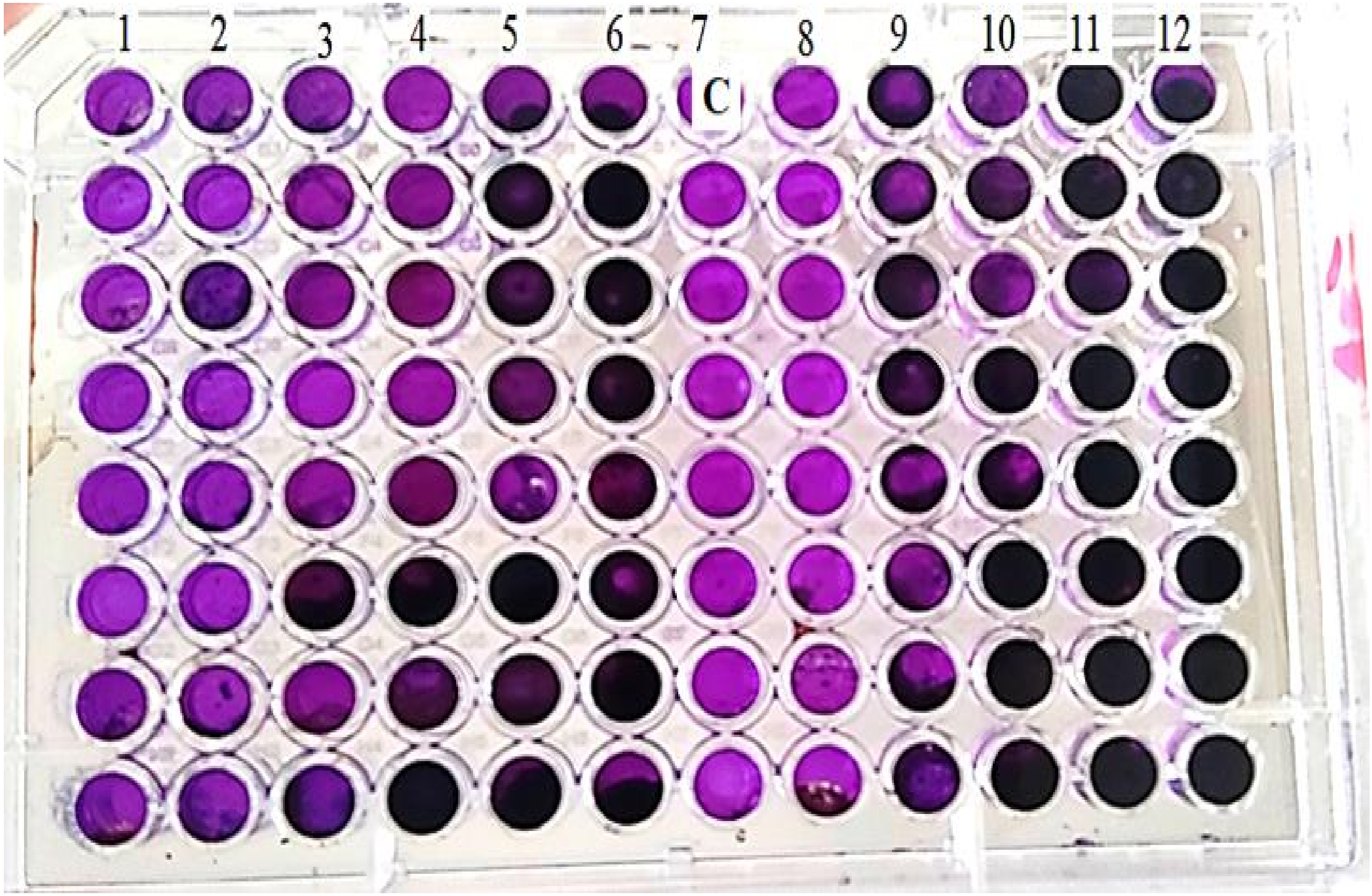

Supplementary figure S4

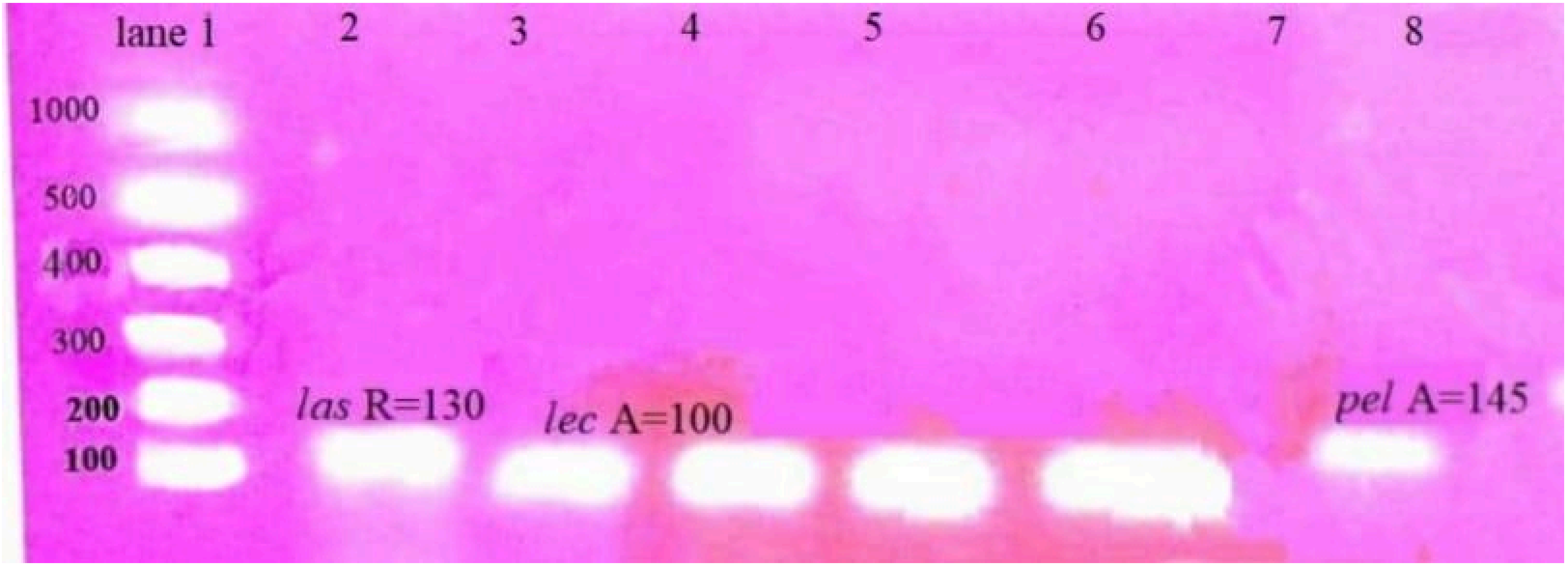

Supplement: Supplementary file 1 — Figures [file 41429_2024_704_MOESM1_ESM.pdf]
