# Supplementary material for: Biofilm formation and antimicrobial resistance pattern of uropathogenic E. coli ST131 isolated from children with malignant tumors
Source: J Antibiot (Tokyo). 2024 Mar 4;77(5):324–30. doi: 10.1038/s41429-024-00704-8 (PMC11058308; doi:10.1038/s41429-024-00704-8)
Supplement: Supplementary file 2 — Supplementary tables [file 41429_2024_704_MOESM2_ESM.docx]

**Supplementary table S1: PCR primers and amplicon size**

| Primer | Sequence | Annealing temperature (^◦^c) | Amplicon size (bp) | Ref. | |
| --- | --- | --- | --- | --- | --- |
| *pab*B | F:TCCAGCAGGTGCTGGATCGT | 63°C | 347 | | 112 |
|  | R:GCGAAATTTTTCGCCGTACTGT |  |  |  |  |
| *trp*A | F:AAAACCGCGCCGCGTTACCT | 63°C | 145 | | 212 |
|  | R:CCAGAAATCGCGCCCGCATT |  |  |  |  |
| *bla*_KPC_ | F:ATGTCACTGTATCGCCGTCT | 58°C | 893 | | 113 |
|  | R:TTTTCAGAGCCTTACTGC CC |  |  |  |  |
| *bla*_IMP_ | F:CTACCG CAG CAGAGTCTT TG | 55 °C | 587 | | 113 |
|  | R:AAC CAG TTT TGC CTTACC AT |  |  |  |  |
| *bla*_VIM_ | F:AGTGGTGAGTATCCGACA G | 55°C | 261 | | 113 |
|  | R:ATGAAAGTGCGTGGAGAC |  |  |  |  |
| *bla_NDM_* | F:GGTTTGGCGATCTGGTTTTC | 58 °C | 621 | | 113 |
|  | R:CGGAATGGCTCATCACGATC |  |  |  |  |
| *las* R | F:AAGTGGAAAATTGGAGTGGAG | 60 ◦c | 130 bp | | 114 |
|  | R:GTAGTTGCCGACGATGAAG |  |  |  |  |
| *lec* A | F:CACCATTGTGTTTCCTGGCGTTCA | 56 ◦c | 100 bp | | 114 |
|  | R:AGAAGGCAACGTCGACTCGTTGAT |  |  |  |  |
| *pel* A | F:AAGAACGGATGGCTGAAGG | 60 ◦c | 148 bp | | 114 |
|  | R: TTCCTCACCTCGGTCTCG |  |  |  |  |

F:forward, R:reverse.

**Supplementary figures legends**

**Supplementary figure S1: Gel images for *pap*B and *trp*A genes.** Lane 1: 100bp ladder, lane 6 *trp* A gene (145bp) and lane 9:  *pap* B gene (347 bp)

**Supplementary figure S2: Gel images of carbapenem-resistance genes.** (**a**) ***bla*_IMP_ and *bla*_VIM_**, Lane 1: 100bp ladder**,** lane 2, 3, 4, 5, 6, 7, 8, 9, 11, 12: *bla*_VIM_ (261bp) and lane 4: *bla*_IMP_(587bp). (**b**) ***bla*_NDM_ and *bla*_KPC_**, Lane 1: 100bp ladder, lane 2, 5: *bla*_KPC_ (893bp) and lane 2, *bla*_NDM_ (621 bp)*.*

**Supplementary figure S3:  Biofilm formation by Micro-titre plate method**. The figure shows crystal violet stained wells of micro-titer plate. Color gradient is different due to different grades of biofilm formation.

**Supplementary figure S4: Gel image of biofilm encoding genes. Lane 1:** 100bp ladder, lane 2: *las R* (130 bp), lane 3, 4, 5 and 6: *lec A* (100 bp) and lane 8:  *pel A* (145bp), bp: base pair.
